# Clipping of unruptured cerebral aneurysms: Are older patients at higher risk?

**DOI:** 10.1007/s00508-021-01887-y

**Published:** 2021-06-15

**Authors:** Fabian Winter, Celia M. Markert, Maximilian Krawagna, Michael Buchfelder, Karl Roessler

**Affiliations:** 1 Department of Neurosurgery, Medical University of Vienna, Währinger Gürtel 18–20, 1090 Wien, Austria; 2 Department of Neurosurgery, Medical University of Erlangen-Nuernberg, Erlangen, Germany; 3 Barmherzige Brueder Klinikum St. Elisabeth, Straubing, Germany

**Keywords:** Neurosurgery, Vascular, Outcome, Elderly, Coiling

## Abstract

**Background:**

The incidence of aneurysms is steadily increasing in older patients due to the aging population. This study compared radiological parameters as well as clinical outcomes between patients younger than 65 years and those over 65 years of age, with special respect to individual treatment options.

**Methods:**

Retrospective data were obtained for patients with cerebral aneurysms at a single academic institution within a 7-year period. Data reviewed included admission protocols, patient charts, operating reports as well as outpatient clinic charts. Aneurysmal characteristics as well as surgical outcome were compared between older patients, defined as patients older than 65 years of age, and a control group of patients younger than 65 years of age. To evaluate and compare individual clinical characteristics various scores including the Hunt and Hess score, the Fisher score, and the Glasgow outcome scale were used.

**Results:**

A total of 347 patients were included in the final analysis. The control group included 290 patients, while 57 patients were in the older patient group. Neither the Hunt and Hess scores nor Fisher scores were significantly correlated to patient age. The Glasgow outcome scale was significantly lower in the older group after clipping of ruptured aneurysms (*p* < 0.000) but not significantly different after clipping of unruptured aneurysms (*p* = 0.793).

**Conclusion:**

Postoperative Glasgow outcome scale scores were not significantly different after clipping of unruptured cerebral aneurysms approximately 1 cm in diameter in older patients compared to the younger age group. Therefore, clipping of unruptured cerebral aneurysms may also be a valuable treatment option for older patients.

## Introduction

The incidence of aneurysms and aneurysmal subarachnoid hemorrhages (SAH) is steadily increasing in elderly patients due to the aging population. Cerebral aneurysms can be treated either by coil embolization or by microsurgical clipping. While both are widely performed, minimally invasive coil embolization is given priority over clipping in elderly patients (65 years and older) [1]; however, coiling is not always technically feasible.

Depending on aneurysmal characteristics, such as size and location but also on patient-specific characteristics such as age, comorbidities and compliance, another option for unruptured aneurysms is observation, especially in very small ones (3 mm or less). Microsurgical clipping compared to coiling is often associated with longer hospital stays due to its invasiveness and higher rates of complications [1, 2]. Thus, clipping of aneurysms for elderly patients is not recommended as first line treatment [2].

This study compared clinical and radiological parameters as well as clinical outcomes between patients younger than 65 years and those over 65 years of age.

## Material and methods

The study protocol was approved by the local ethics committee of the University of Erlangen, Germany, and was in line with the Helsinki Declaration of Human Rights. Retrospective data were obtained for patients with cerebral aneurysms at the Department of Neurosurgery at the University of Erlangen within a 7-year time period. Data reviewed included admission protocols, patient charts, operating reports, intensive care unit charts, discharge protocols, preoperative and postoperative imaging protocols, as well as outpatient clinic charts obtained during follow-up visits.

Aneurysmal characteristics reviewed on preoperative imaging were compared between elderly patients, defined as patients older than 65 years of age, and a control group of patients younger than 65 years of age. In both groups, postoperative outcome of ruptured aneurysms leading to subarachnoid hemorrhages and unruptured aneurysms were grouped and compared, especially with respect to microsurgical clipping.

Aneurysmal subarachnoid hemorrhage characteristics were classified using the Hunt and Hess classification (HH) as well as the Fisher score [3, 4]. In addition, this study used the Glasgow outcome scale (GOS) to report clinical outcome after surgery by the objective degree of recovery [5]. The 5‑grade scale allows a prediction of rehabilitation to return to everyday life.

## Statistical analysis

Microsoft Excel (Microsoft Corporation, Redmond, WA, USA) and SPSS (v25, IBM, IBM, Armonk, NY, USA) were used to collect and analyze data. Regression and correlation analyses were carried out utilizing the Mann-Whitney U-test. Descriptive statistics were performed to depict mean values. For small numbers of cases Fisher’s exact test was used for nominal variables. The Spearman correlation was used for comparing GOS values. A *p* value < 0.05 was considered statistically significant.

## Results

**Fig. 1:**
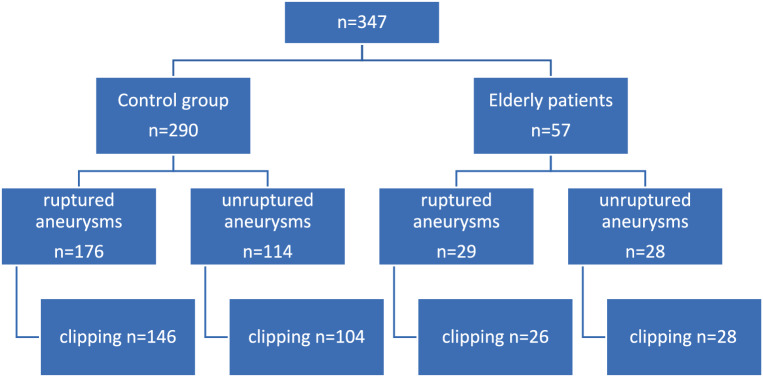
Patient inclusion with respect to microsurgical clipping in the control and elderly groups

A total of 347 patients were included in the final analysis. The control group included 290 patients, with 197 females and 93 males and a mean age of 49.7 ± 8.8 years. The group of elderly patients included 57 patients, with 43 females and 14 males and a mean age of 70.9 ± 4.4 years. Patients were followed up for a mean duration of 9.8 ± 13.8 months (Fig. 1).

### Hunt and Hess and Fisher scores

The HH score of ruptured aneurysms was not significantly different between the control group (mean 2.4) and the elderly patients (mean 3.0, *p* = 0.067). The appearance of subarachnoid hemorrhage was not significantly different between the control group (Fisher score of mean 3.1) and the elderly patients (Fisher score of mean 3.1, *p* = 0.574) (Table 1).

**Table 1:** Patient demographics and clinical scores of the present study

	Control patients *n* = 290		Elderly patients *n* = 57	
	Ruptured	Unruptured	Ruptured	Unruptured
	*n* = 176	*n* = 114	*n* = 29	*n* = 28
Age (years)	48.4 ± 8.9	51.8 ± 8.3	72.4 ± 5.0	69.4 ± 3.2
Female	114	83	21	22
Male	62	31	8	6
Size (mm)	6.6 ± 3.7	6.8 ± 4.2	6.1 ± 3.9	6.4 ± 2.9
*HH*				
Grade I	59	–	5	–
Grade II	32	–	6	–
Grade III	42	–	7	–
Grade IV	28	–	8	–
Grade V	14	–	3	–
*Fisher*				
Grad I	4	–	0	–
Grad II	39	–	7	–
Grad III	71	–	10	–
Grad IV	60	–	12	–
*GOS*				
Grad I	12	0	5	0
Grad II	6	0	6	1
Grad III	17	0	8	2
Grad IV	52	24	3	6
Grad V	89	90	7	19

### Glasgow outcome scale

**Fig. 2:**
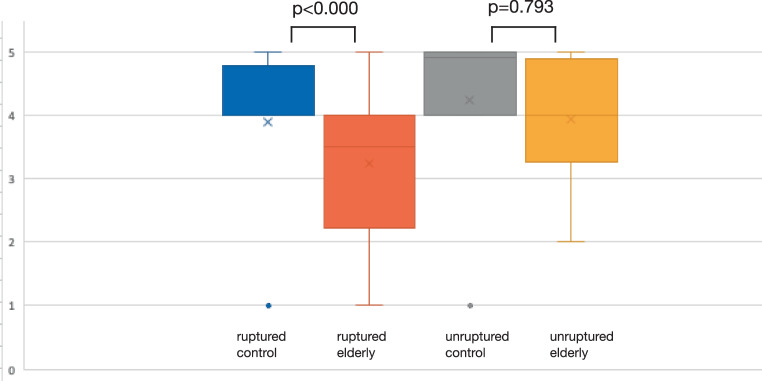
Glasgow outcome scale scores for unruptured and ruptured aneurysms in the control and elderly group

The GOS was negatively correlated to the HH throughout all age groups (*p* < 0.001, r = −0.431). The GOS for ruptured aneurysms treated with microsurgical clipping of the control group was 4.1 ± 1.2, while it was 4.5 ± 0.8 in patients after minimally invasive coil embolization. This was significantly better compared to the elderly patient group (*p* < 0.000) as it was 2.9 ± 1.4 after microsurgical clipping, and 4.0 ± 1.0 after coil embolization. The GOS for unruptured aneurysms was 4.8 ± 0.4 in the control group and 4.5 ± 0.8 in the elderly patient group after microsurgical clipping. Therefore, the GOS was not significantly different between the control and the elderly patient group after microsurgical clipping of unruptured aneurysms (*p* = 0.793) (Fig. 2). Furthermore, the GOS was significantly better throughout all age groups after treatment of unruptured aneurysms compared to ruptured aneurysms (*p* < 0.000).

### Control group

Ruptured aneurysms were treated with microsurgical clipping in 146 patients, while 24 patients underwent minimally invasive coil embolization. Treatment was performed after a mean of 3.2 days after onset of symptoms. The size of the aneurysm was 6.6 ± 3.7 mm in diameter. Unruptured aneurysms were diagnosed in 114 patients. The size of the aneurysms was 6.8 ± 4.2 mm in diameter. The majority (104 patients) underwent microsurgical clipping. Altogether, aneurysm sizes were not significantly different between the investigated groups.

In the control group, the majority of ruptured aneurysms were located at the anterior communicating artery (42.6%), followed by aneurysms located at the middle cerebral artery (36.4%) and various locations of the posterior circulation (9%) including the posterior communicating artery, posterior inferior cerebellar artery, superior cerebellar artery, and basilar artery. In the case of unruptured aneurysms the location in the control group was mainly at the middle cerebral artery (47.4%), followed by the anterior communicating artery (26.3%) and aneurysms located in the posterior circulation (5.3%).

### Elderly group

Ruptured aneurysms were treated with microsurgical clipping in 26 patients, while 3 patients underwent minimally invasive coil embolization in the elderly group. Treatment was performed after a mean of 5.79 days after onset of symptoms. The size of ruptured aneurysms was 6.1 ± 3.9 mm in diameter and was not significantly different between the two groups (*p* = 0.440).

Unruptured aneurysms were clipped in all 28 patients. The size of the aneurysms was 6.4 ± 2.9 mm in diameter and was also not significantly different between the two groups (*p* = 0.813). Treatment was performed after a mean of 36.3 ± 87.1 days after first diagnosis.

In the elderly group, the majority of ruptured aneurysms were located in the middle cerebral artery (41.4%), followed by anterior communicating artery aneurysms (24.1%), while 13% were located in the posterior circulation. For unruptured aneurysms the location varied from aneurysms in the anterior communicating artery (46.4%), middle cerebral arteries in (32.1%) and aneurysms in the posterior circulation (7.1%).

## Discussion

We found that the GOS of patients older than 65 years was not significantly lower compared to younger patients after microsurgical clipping of unruptured aneurysms; however, clipping of ruptured aneurysms after SAH in elderly patients was significantly worse with lower GOS compared to the control group of patients younger than 65 years old.

Incidences of surgical mortality and morbidity after microsurgical clipping of an intracerebral aneurysm have been reported between 0–5.2% and 5.6–26.8%, respectively, regardless of age, comorbidities and ruptured versus unruptured aneurysms [6–9]. Furthermore, the incidence of cerebral aneurysms and thus the incidence of subarachnoid hemorrhage caused by ruptured aneurysms increases with patient age [10]. Therefore, treatment options for cerebral aneurysms in elderly patients are widely discussed [2, 6, 9, 11]. The number of elderly patients included is comparable to other published data [9, 12, 13]. Mori et al. demonstrated that surgical outcomes in elderly patients were not significantly different from those in the nonelderly patients [9]; however, they limited their findings to aneurysms in the anterior circulation for relatively small aneurysms. The present study did not distinguish between small and large aneurysms. In addition, all locations of cerebral aneurysms were included in this study. Aneurysm location nor size were significantly different with age; however, the majority of aneurysms were also located in the anterior circulation. In contrast to those findings Brinjikji et al. and Wiebers et al. both stated that advanced age was a strong predictor for poor outcome in both clipping and coiling cohorts but especially for elderly patients undergoing clipping of an aneurysm [6, 11].

Frailty is common in elderly patients. Thus, risk of postoperative complications and increased risks for morbidity, longer hospitalization and mortality have been reported by numerous authors [11, 14, 15]. The present study stands in concordance with those findings showing worse GOS scores, and therefore poor postoperative outcome in elderly patients after microsurgical clipping of ruptured aneurysms. This can be explained due the fact that subarachnoid hemorrhage in elderly patients itself is a limiting component in postinterventional outcomes. In contrast, we found equal outcomes in the elderly compared to younger patients after clipping of unruptured aneurysms, which means that surgical clipping is a valuable alternative in elderly patients when coiling is not possible [9].

## Limitations

Due to its retrospective design, data acquisition and the statistical power were limited due to uneven cohort sizes. In addition, selection bias with respect to patients who underwent clipping versus coiling cannot be ruled out; however, patient demographics and the number of included patients are comparable to other published series.

## Conclusion

Postoperative GOS scores are not significantly worse after clipping of unruptured cerebral aneurysms with a diameter of approximately 1 cm in elderly patients compared to younger patients. Therefore, clipping of unruptured cerebral aneurysms is also a valuable treatment option for elderly patients.
